# Effect of intragastric versus small intestinal delivery of enteral nutrition on the incidence of pneumonia in critically ill patients: a complementary meta-analysis

**DOI:** 10.1186/cc13978

**Published:** 2014-07-08

**Authors:** Wan-Jie Gu, Jing-Chen Liu

**Affiliations:** 1 Department of Anaesthesiology, the First Affiliated Hospital, Guangxi Medical University, Nanning 530021, Guangxi, China

## 

As intensive care specialists with a long-time interest in the relative pros and cons of intragastric and small intestinal delivery of enteral nutrition in critically ill patients, we greatly enjoyed reading the excellent meta-analysis performed by Deane and colleagues based on randomized clinical trials (RCTs) [1]. The analysis demonstrated small bowel feeding was associated with a reduced risk of pneumonia (relative risk (RR) 0.75; 95% CI 0.60 to 0.93; *P* = 0.01). The authors concluded that use of small intestinal feeding may reduce the incidence of ICU-acquired pneumonia. Although this result has significant clinical implications, one important issue should be addressed.

Based on the same articles used by Deane and colleagues [1], we performed a complementary meta-analysis to reappraise the effect of intragastric versus small intestinal delivery of enteral nutrition on the incidence of pneumonia. The data extracted from the 12 RCTs were stratified by population (trauma versus non-trauma population). It was shown that small bowel feeding was associated with a reduction in the incidence of pneumonia in trauma (four RCTs; RR 0.67; 95% CI 0.52 to 0.87; *P* = 0.003; references [2-5] in the article by Deane and colleagues), but no reduction in non-trauma population (eight RCTs; RR 0.86; 95% CI 0.58 to 1.26; *P* = 0.43; references [6-13] in the article by Deane and colleagues) (Figure 1).

**Figure 1:**
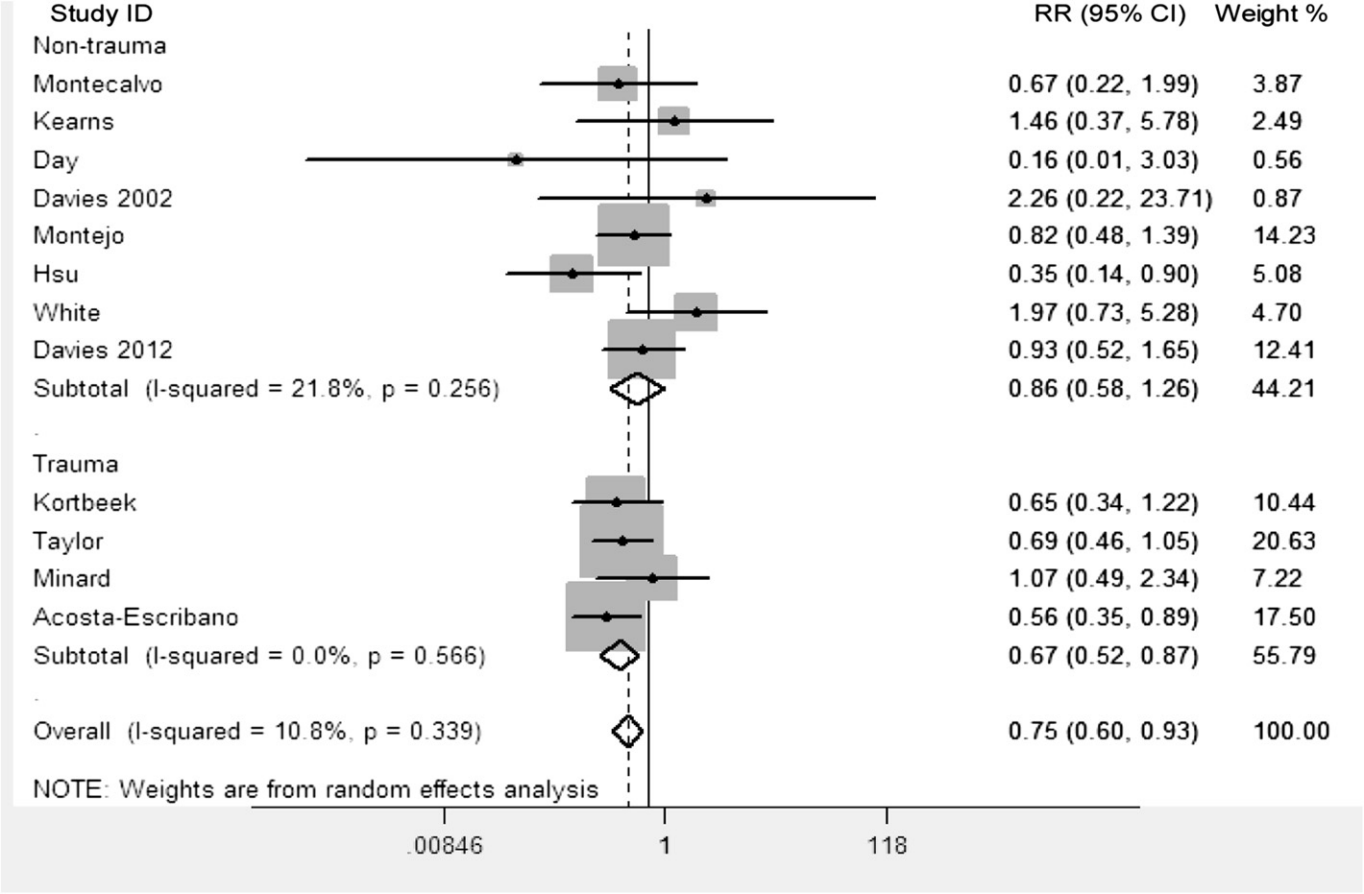
**The 12 studies reporting the incidence of pneumonia used for the complementary meta-analysis.** RR, relative risk (small bowel feeding versus gastric feeding).

What is noteworthy is that the rate of pneumonia reported in the trauma population was approximately two to three times higher than that in the non-trauma population (trauma versus non-trauma population: 132/293 (45.1%) versus 131/683 (19.2%)). This potentially further limits the generalizability of the conclusions from the study of Deane and colleagues.

## Authors' response

Adam M Deane, Rupinder Dhaliwal, Andrew Day, Emma J Ridley, Andrew R Davies and Daren K Heyland

In response to our systematic review [1] it has been suggested that the reduction in pneumonia observed with small intestinal delivery occurs only in patients following trauma.

Following traumatic injury, patients are at risk of developing enteral feed intolerance [2], and it is intuitive that these patients will have greater capacity to benefit from small intestinal feeding [3]. Furthermore, injuries frequently demand that patients remain without head elevation, positioning that increases the risk of developing pneumonia [4]. There is, therefore, a strong biological plausibility that this population will benefit from small intestinal feeding to a greater extent than other populations.

However, inferences about subgroup effects in systematic reviews should be made cautiously, as comparisons must be consistent between and within studies [5]. Several of the studies included by Gu and Liu as non-trauma population were studies performed in mixed medical/surgical ICUs. Indeed, several of these studies actually included a proportion of patients following traumatic injuries. On detailed analysis of demographic data from the Montecalvo (reference [30] in our study), Davies 2012 (reference [21] in our study), Davies 2002 (reference [32] in our study) and White (reference [18] in our study) studies (Figure 1), 39%, 37%, 27% and 8%, respectively, of enrolled patients were admitted following trauma and were included in the complementary meta-analysis but were grouped in the non-trauma population.

While this complementary meta-analysis is of interest, it may be explained by differences between studies and/or by chance. Accordingly, we suggest that further analysis, using individual patient data meta-analysis, is required before the observation by Gu and Liu can be considered hypothesis generating.

## Abbreviations

RCT: randomized clinical trial; RR: relative risk.

## Competing interests

The authors declare that they have no competing interests.

## Authors' contributions

WJG and JCL conceived the study, participated in the design, collected the data, and drafted the manuscript. All authors read and approved the final manuscript.
